# Actin filaments disruption and stabilization affect measles virus maturation by different mechanisms

**DOI:** 10.1186/1743-422X-10-249

**Published:** 2013-08-02

**Authors:** Erik Dietzel, Larissa Kolesnikova, Andrea Maisner

**Affiliations:** 1 Institute of Virology, Philipps University of Marburg, Hans-Meerwein-Str 2, Marburg, D-35043, Germany

**Keywords:** Measles virus, Assembly, Budding, Jasplakinolide, Actin dynamics

## Abstract

**Background:**

Cytoskeletal proteins are often involved in the virus life cycle, either at early steps during virus entry or at later steps during formation of new virus particles. Though actin filaments have been shown to play a role in the production of measles virus (MV), the importance of actin dynamics for virus assembly and budding steps is not known yet. Aim of this work was thus to analyze the distinctive consequences of F-actin stabilization or disruption for MV protein trafficking, particle assembly and virus release.

**Results:**

MV infection studies in the presence of inhibitors differently affecting the actin cytoskeleton revealed that not only actin disruption but also stabilization of actin filaments interfered with MV particle release. While overall viral protein synthesis, surface expression levels of the MV glycoproteins, and cell-associated infectivity was not altered, cell-free virus titers were decreased. Interestingly, the underlying mechanisms of interference with late MV maturation steps differed principally after F-actin disruption by Cytochalasin D (CD) and F-actin stabilization by Jasplakinolide (Jaspla). While intact actin filaments were shown to be required for transport of nucleocapsids and matrix proteins (M-RNPs) from inclusions to the plasma membrane, actin dynamics at the cytocortex that are blocked by Jaspla are necessary for final steps in virus assembly, in particular for the formation of viral buds and the pinching-off at the plasma membrane. Supporting our finding that F-actin disruption blocks M-RNP transport to the plasma membrane, cell-to-cell spread of MV infection was enhanced upon CD treatment. Due to the lack of M-glycoprotein-interactions at the cell surface, M-mediated fusion downregulation was hindered and a more rapid syncytia formation was observed.

**Conclusion:**

While stable actin filaments are needed for intracellular trafficking of viral RNPs to the plasma membrane, and consequently for assembly at the cell surface and prevention of an overexerted fusion by the viral surface glycoproteins, actin dynamics are required for the final steps of budding at the plasma membrane.

## Background

Measles virus (MV) is a prototype member of the Morbillivirus genus in the family *Paramyxoviridae*. In virus particles, the negative-stranded RNA genome is encapsidated by the N, P and L proteins, and this ribonucleocapsid (RNP) is surrounded by a lipid bilayer. The two surface glycoproteins, the hemagglutinin H and the fusion protein F, protrude from the viral envelope. The matrix protein (M) is located at the inner surface of the lipid bilayer tethering the RNP to the envelope. Due to its interaction with the glycoproteins and the RNPs, the M protein is essential for MV assembly and particle formation. M binding to the cytoplasmic tails of the glycoproteins at the surface of infected cells is furthermore required to downregulate H/F-mediated cell-to-cell fusion of infected and neighboring uninfected cells [1-5].

The actin network is primarily associated with mechanical stability, cell motility and cell contraction. It is also important for chromosome movement during mitosis and for internal transport, particularly near the plasma membrane. Cargos can be transported either by riding on myosin motors along actin filaments or by pushing forces exerted by actin as it undergoes polymerization [6]. Cytoskeletal actin not only has a central function in cell physiology but is also an essential component involved in the replication of many RNA and DNA viruses. The molecular mechanisms underlying this important host-virus interaction, however, are extremely diverse [7]. For MV, several reports have shown that actin is involved in virus maturation at the plasma membrane. This idea was initially based on the findings that actin was identified as an internal component of MV particles [8,9] and co-caps with MV H on infected cells [10]. There is further ultrastructural evidence that actin filaments take part in the process of budding and protrude into viral buds [7,8]. Very recently, it was furthermore proposed that F-actin associates with the MV M protein altering the interaction between M and H, hereby modulating MV cell-cell fusion and assembly [11].

Though there is conclusive evidence that intact actin filaments are important for MV replication, it is not yet defined if a stable actin cytoskeleton is sufficient, or if actin dynamics are required. Aim of this study was thus to analyze the effects of actin-disrupting and actin-stabilizing drugs to define if actin filaments as structural components or rather actin dynamics and treadmilling are essential for MV maturation. Actin treadmilling is a process in which actin filament length remains approximately constant but actin monomers preferentially join with the barbed ends and dissociate from the pointed ends of filaments. This oriented renewal of actin within microfilaments causes a treadmilling involving both, actin monomers and actin-binding proteins. Jasplakinolide (Jaspla) is a cyclic peptide isolated from a marine sponge that binds to and stabilizes filamentous actin, inducing a blockade of actin treadmilling [12,13]. In contrast to Jaspla, Cytochalasin D (CD), a fungal metabolite, serves as an actin capping compound that binds to the barbed (+) end of actin filaments and shifts the polymerization-depolymerization equilibrium towards depolymerization of F-actin [14].

With our studies on MV replication in the presence of CD and Jaspla, we show that defects in actin polymerisation and defects in actin depolymerisation can both interfere with late virus assembly and budding steps without impairing overall viral protein synthesis, cell-associated infectivity or the surface transport of the MV glycoproteins. Most interestingly, the underlying mechanism of interference with late MV maturation steps by CD and Jaspla differs principally. While intact actin filaments that can be disrupted by CD treatment are required for M-RNP cotransport from the cytoplasm to the plasma membrane, actin dynamics at the cytocortex blocked by Jaspla are necessary for later steps in virus maturation at the plasma membrane. Supporting our finding that actin disruption blocks M-RNP transport to the plasma membrane, cell-to-cell spread of MV infection was enhanced upon CD treatment. Due to the lack of M-glycoprotein-interaction at the cell surface, M-mediated fusion downregulation is hindered and a more rapid syncytia formation is observed in CD-treated cells.

## Results

### Actin disruption and stabilization affect virus release without influencing the amount of cell-associated infectivity

**Figure 1:**
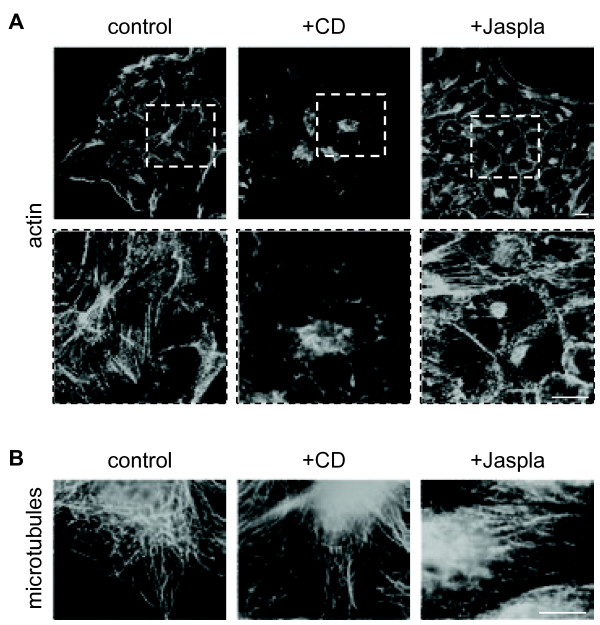
**Influence of F-actin disruption and stabilization on the actin cytoskeleton and microtubules.** MDCK cells were incubated with the inhibitors (+CD, +Jaspla) which were added at 12 h after seeding. **(A)** For actin staining at 48h p.s., cells were fixed with PFA and permeabilized with Triton X-100. F-actin was detected by phalloidin-FITC. Images were obtained using a confocal laser scanning microscope (Zeiss LSM510). Lower panels show higher magnification of the boxed areas in upper panels. **(B)** For microtubule staining at 48 h p.s., cells were fixed with methanol/acetone and incubated with a monoclonal anti-α-tubulin antibody and fluorophore-conjugated secondary antibodies. Images were obtained using a fluoresence microscope (Zeiss Axiovert 200 M). Bars 10 μm.

**Figure 2:**
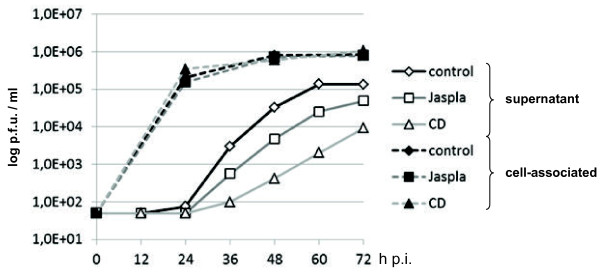
**Effect of F-actin disruption and stabilization on virus release and cell-associated infectivity.** MDCK cells were infected with MV at an MOI of 10. Inhibitors were added at 12 h p.i. Supernatants were taken in 12 hour intervals to titrate cell-free virus and cells were harvested at 24, 48 and 72 h p.i. To titrate cell-associated infectivity, virus titers were quantitated by plaque assay. Values plotted represent mean results of two independent experiments.

To analyze the importance of the actin polymerization and depolymerization on MV infection, we quantitated the release of infectious MV particles and the cell-associated infectivity in the absence and presence of 4 μM Cytochalasin D (CD) or 100 nM Jasplakinolide (Jaspla). To visualize the effect and the specificity of the two inhibitors, actin filaments and microtubules in non-infected cells treated with CD or Jaspla were either stained with Phalloidin-FITC or with an anti-tubulin antibody and an AlexaFluor (AF)488-labelled secondary antibody, respectively. Actin stress fibers and subcortical actin were readily stained in control cells (Figure 1A). As CD causes actin disruption by binding to the barbed plus ends of actin filaments, treatment with 4 μM CD led to the disappearance of stress fibers and the actin-dense cytocortex. Actin was rather found in punctate structures. In contrast to CD, the effect of the actin filament stabilizing drug Jaspla was evident by the formation of perinuclear F-actin aggresomes [15] (Figure 1A). None of the inhibitors affected the filamentous structure of microtubules (Figure 1B) demonstrating that their effect was specific for actin and not due to a general breakdown of the cell cytoskeleton. To exclude that cytotoxic effects due to inhibitor treatment affect the analyses, cell viability was tested at 48 h p.i. by incubating live cells with propidium iodide (PI), a membrane impermeant DNA dye, followed by cell permeabilization and nuclear DAPI staining. Control and inhibitor treated cells were negative for PI staining, confirming that the cells were viable and that the plasma membrane was still intact (data not shown). To monitor MV infection in the presence of the inhibitors, MDCK cells were infected with MV at an MOI of 10. At 12 h p.i., inhibitors were added and the cells were further incubated up to 72 h p.i.. Aliquots from the cell supernatants were taken every 12 h to determine the cell-free virus titers. To determine the cell-associated infectivity, cell lysates were prepared by one freeze-thaw cycle and were titrated by plaque assay on confluent Vero cells. Cell disruption by freezing and thawing allows isolation of intracellular viral RNPs not yet finally assembled or budding at the plasma membrane. Figure 2 shows that this cell-associated infectivity was not reduced by CD or Jaspla treatment suggesting that inhibitor treatments did not principally diminish the number of infected cells or the production of infectious RNPs and viral proteins. In contrast, virus titers in the supernatants were clearly reduced. In CD- and Jaspla-treated cells, virus release at 48 h p.i. was reduced by 99% (2 log steps) and 80% (1 log step), respectively. A similar reduction of virus release by CD and Jaspla treatment was observed in Vero cells (data not shown). In agreement with the described reversibility of the actin inhibitors, the effects of the inhibtors on the production of viruses were reversible. When we removed the inhibitors at 48 h p.i., final virus titers almost reached control levels within 24 h. While control cells released 5.1×10^5^ p.f.u./ml between 48 and 72 h p.i., virus yield produced between the time of CD and Jaspla removal and 72 h p.i. increased up to 1.7×10^5^ p.f.u./ml and 3.5×10^5^ p.f.u./ml, respectively.

### Viral protein expression is not altered upon inhibitor treatment

**Figure 3:**
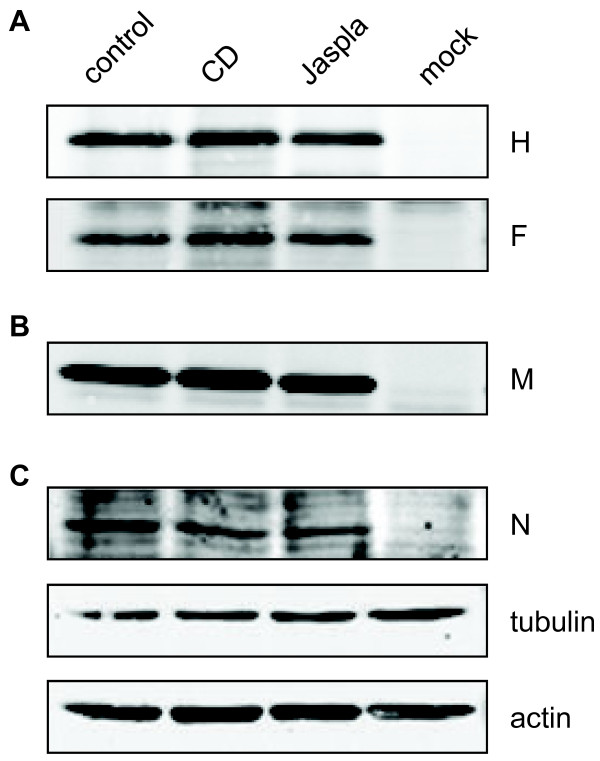
**Viral protein expression upon F-actin disruption and stabilization.** MDCK cells were infected with MV at an MOI of 10 (control, CD, Jaspla), or left uninfected (mock). Inhibitors were added at 12 h p.i.. At 48 h p.i., cells were surface biotinylated and subsequently lysed in RIPA buffer. **(A)** To determine the amount of MV glycoproteins expressed on the cell surface, H and F were immunprecipitated from cell lysates with specific monoclonal antibodies. Samples were then separated by SDS-PAGE, transferred to nitrocellulose and probed with AF680-conjugated streptavidin. **(B)** M protein was immunoprecipitated from cell lysates using an anti-M specific antibody. Precipitates were subjected to western blot analysis using M-specific primary antibodies and AF680-conjugated secondary antibodies. **(C)** For N, tubulin and actin staining, lysates were directly subjected to SDS-PAGE, blotted to nitrocelluose and incubated with specific primary antibodies and fluorophore-conjugated secondary antibodies. Blots were scanned with a Li-Cor Odyssey IR system.

To determine if the reduced virus release upon CD and Jaspla treatment is due to an overall change in viral protein expression levels or an altered surface expression of the viral glycoproteins, MV-infected and inhibitor-treated cells were surface biotinylated. For this purpose, the cell membrane impermeant reagent S-NHS-biotin was added at 48 h p.i., the time point at which control virus release was almost maximal and the inhibitor-induced reduction of virus release was most pronounced. After biotin labelling, cells were lysed and the MV glycoproteins H and F were immunoprecipitated by specific antibodies. Precipitates were separated by SDS-PAGE and transferred to nitrocellulose. Biotinylated H and F proteins were then detected using AF680-conjugated streptavidin (Figure 3A). Since the viral M protein is an intracellular protein and thus not surface biotinylated, it was detected after immunoprecipitation by Western blot analysis (Figure 3B). The viral N protein and cellular tubulin and actin were directly detected in the cell lysates by western blot analysis (Figure 3C). Figure 3 shows no substantial differences in the protein amounts in inhibitor-treated cells compared to control cells. We thus conclude that neither changes in the overall protein expression levels, nor a decreased surface expression of the viral glycoproteins can be responsible for the observed reduction in virus particle formation upon actin filament disruption or stabilization.

### Cytochalasin D but not Jasplakinolide treatment leads to retention of M-RNP complexes in the cytoplasm

**Figure 4:**
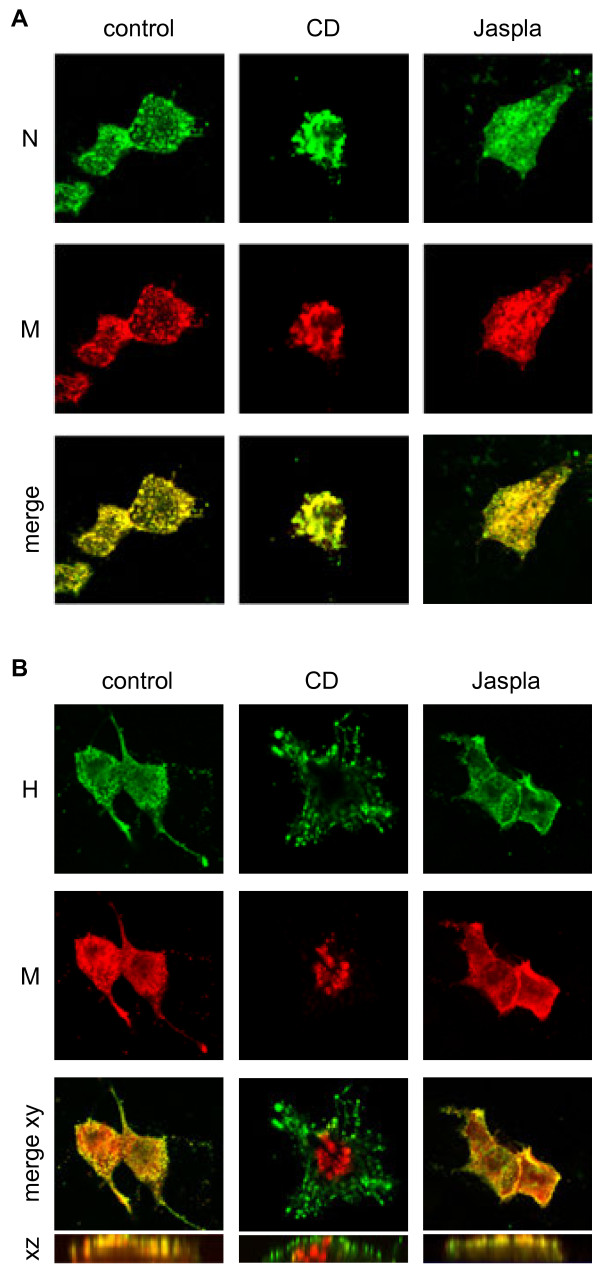
**Effect of F-actin disruption and stabilization on viral protein distribution.** MDCK cells were infected with MV at an MOI of 10. To prevent fusion, an inhibitory peptide (FIP) was added. Inhibitors (CD, Jaspla) were added at 12 h p.i.. **(A)** For N and M protein co-staining, cells were fixed and permeabilized with methanol/acetone. N was detected by a polyclonal rabbit antiserum and AF488-conjugated secondary antibodies. M was stained using a monoclonal antibody (MAB8910) and AF568-conjugated secondary antibodies. **(B)** For H and M co-staining, cells were fixed with PFA, permeabilized with Triton X-100 and H was detected with a monoclonal antibody (K83) and AF488-conjugated secondary antibodies. Afterwards, the M protein was stained with an AF555-labelled monoclonal anti-M antibody (MAB8910). xy sections of the merged images (merge xy) and a side view (xz) are shown. Images were recorded with a confocal laser scanning microscope (Zeiss LSM510). Magnification 630x.

To further elucidate which assembly step is inhibited by actin disruption or stabilization, we analyzed the influence of inhibitor treatment on the intracellular localization of the viral proteins. For this purpose, co-immunostaining of M and H protein or M and N protein was performed. At 48 h p.i., cells were fixed and permeabilized and the proteins were detected with specific primary antibodies and AF488- (N protein) or AF568-labelled (M protein) secondary antibodies. As shown in Figure 4A, M and N proteins (RNPs) colocalized almost completely in control and inhibitor-treated cells. However, while M-RNP complexes in control and Jaspla-treated cells were predominantly located at the plasma membrane, the complexes accumulated in the cytoplasm after CD treatment. The intracellular localization of the proteins was confirmed by altering the gain in the confocal image to identify the cell limits. To corroborate the lack of M transport to the cell surface, M-H co-staining was performed. For this, cells were fixed and permeabilized with Triton X-100 and the H protein was detected by an H-specific antibody and AF488-conjugated secondary antibodies. The M protein was detected with an AF555-labelled monoclonal anti-M antibody. Supporting the idea of a defective M-RNP transport upon CD treatment, H and M colocalized markedly in control and Jaspla-treated cells whereas M was found in large intracellular patches in CD-treated cells (Figure 4B). The lack of H and M colocalization is clearly seen in the in the side view (vertical xz section) of the merged image. This result indicates that intact actin filaments are essentially required for M-RNP surface transport. Reduced virus release upon CD treatment is therefore concluded to be due to a defective budding as a result of a reduced amount of viral M-RNP complexes present at the plasma membrane. Interestingly, the reduced particle release upon Jaspla treatment does not appear to be linked to a defective M-RNP transport to the plasma membrane.

### Actin disruption by Cytochalasin D increases cell-to-cell fusion

**Figure 5:**
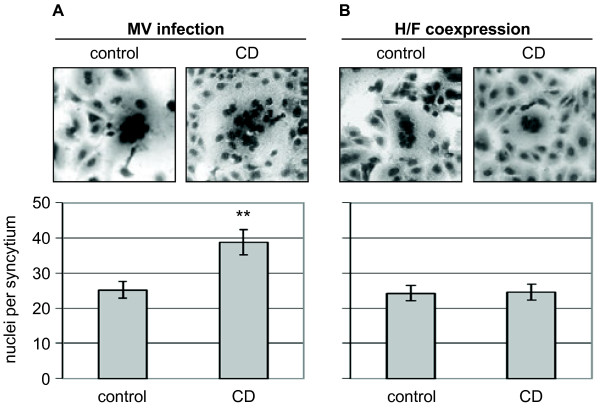
**Influence of cytochalasin D treatment on MV glycoprotein-mediated cell-to-cell fusion. (A)** MDCK cells were infected with MV at an MOI of 10 in the presence of a fusion-inhibitory peptide (FIP). CD was added at 12 h p.i., and cells were washed, detached and mixed with non-infected and non-inhibitor-treated Vero cells at 48 h p.i.. 5 h later, syncytium formation was visualized by Giemsa staining. **(B)** Cells were cotransfected in the presence of FIP with plasmids encoding the MV glycoproteins H and F. CD was added at 6 h post transfection, and cells were washed and detached at 24 h post transfection. 5 h after mixing with non-treated Vero cells, syncytia formation was visualized by Giemsa staining. In the upper panels, a syncytium is exemplarily shown. Fusion was quantified as described in the methods and is shown in the lower panels. Error bars indicate standard error of the mean (SEM). ** P < 0.01.

Actin disruption did not influence surface expression of the viral glycoproteins but led to retention of M-RNP complexes in the cytoplasm. As it is known that M downregulates cell-to-cell fusion by interacting with the cytoplasmic tails of the viral glycoproteins at the plasma membrane [1-3], we wanted to assess if syncytium formation of MV-infected MDCK cells is affected by the inhibitor treatment. Since MDCK cells do not fuse rapidly and fusion progression might be further hindered by cell-morphological alterations due to the inhibitor treatment, fusion capacity was monitored by detaching the infected, inhibitor-treated MDCK cells with accutase and subsequent mixing with a Vero cell suspension. Non-infected and non-inhibitor treated Vero cells serve here as “fusion indicator cells”. Cell mixtures were co-cultured for 5 h and syncytia formation was visualized by Giemsa staining. As shown in Figure 5A, MV-infected cells showed an increased fusion activity after CD treatment. This clearly supports our idea that CD-mediated retention of the M protein in the cytoplasm resulted in a reduced M-glycoprotein interaction at the plasma membrane, and thus to a reduced fusion downregulation by M. Since it has been proposed that actin structures can also restrict fusion-pore extension [16,17], we wanted to rule out that the observed increased syncytia formation upon CD treatment might be simply due to the disruption of the actin cytocortex facilitating expansion of the fusion pores. We therefore analyzed the effect of CD treatment on MV glycoprotein-mediated cell-to-cell fusion in the absence of M and any virus infection. For this purpose, we coexpressed H and F proteins in MDCK cells, incubated the cells either in the absence or presence of CD for 18 h, and performed the fusion assay as described above. In contrast to the infection, H and F cotransfected cells did not show enhanced fusion capacity after CD-mediated actin disruption. We thus conclude that the increase of the relative fusion after CD treatment in MV-infected cells is due to a reduced M-mediated fusion downregulation, and is therefore the consequence of the defective M surface transport in cells with a disrupted actin cytoskeleton.

### Actin stabilization by Jasplakinolide affects late MV maturation steps

**Figure 6:**
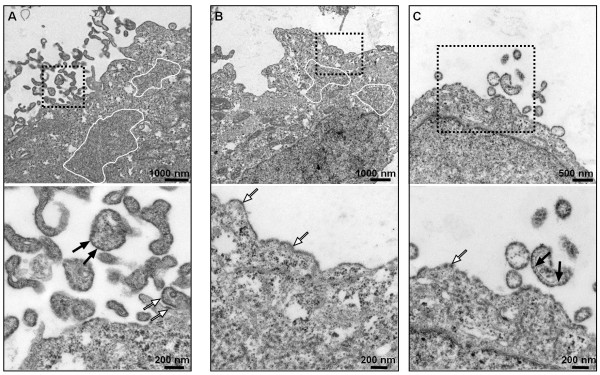
**Effect of F-actin stabilization on MV budding.** Electron microscopic analysis of ultrathin sections of MV-infected Vero cells either left without treatment **(A**, control**)**, or treated with 0.2 μM Jaspla at 12 h p.i. **(B** and **C)**. White-line marked areas show accumulations of RNPs in the cytoplasm. Black-boxed areas on upper panels are shown at higher magnifications in lower panels. Black arrows indicate RNPs inside viral particles; white arrows indicate RNPs beneath the plasma membrane.

Jaspla treatment reduced virus release by more than 80% without affecting viral protein synthesis or downregulating the surface transport of viral glycoproteins and M-RNP complexes. Thus, very late budding steps at the plasma membrane appeared to be affected by actin stabilization. To address this question, we performed ultrastructural analyses of MV-infected Vero cells, in which 200 nM Jaspla treatment resulted in reduction of virus release into the supernatant by 83% (data not shown). For the EM analysis, MV-infected cells were treated with Jaspla at 12 h p.i.. After fixation at 48 h p.i., cells were processed for EM analysis. While in control cells, virus particles and bud formation were abundantly found (Figure 6A), detection of virus buds and cell-free particles was much more restricted in Jaspla-treated cells (Figure 6B, 6C). RNPs were highly concentrated and aligned at the plasma membrane. However, budding events are rarely seen in Jaspla-treated cells (Figure 6B, 6C, lower panels). This clearly confirms that actin stabilization does not block delivery of M-RNP complexes to the plasma membrane but rather prevents the formation of viral buds and the final pinching off.

## Discussion

Consistent with an earlier report using Cytochalasin B [18], we found that CD inhibited MV replication. Actin inhibitors specifically seem to block late virus assembly and budding steps, because inhibition of earlier replication steps such as RNA synthesis would be reflected in decreased overall virus protein expression levels and reduced titers of both, released virus and cell-associated infectivity. After actin disruption by CD, M-RNP complexes were retained in the cytoplasm implicating a role for intact actin filaments in M-RNP transport. Consistent with M acting as a fusion downregulator, we found an increased cell-to-cell fusion after actin disruption due to the lack of M-glycoprotein interaction at the plasma membrane. Similar to actin disruption, actin stabilization by Jaspla led to decreased cell-free virus titers. However, the underlying molecular mechanism is clearly different. Block of actin dynamics by Jaspla did not result in the intracellular retention of M-RNP complexes. Immunofluorescence and electron microscopic observations rather indicate a defect in bud formation and subsequent pinching-off.

Actin filaments (disrupted by CD) but not actin treadmilling (blocked by Jaspla) are needed for transport of M and MV nucleocapsids from the cytoplasm to the cell surface. Yet, actin dynamics seem to play a role in budding of mature virions at the plasma membrane. Even if an early report in chronically MV-infected cells had suggested that RNP but not the M transport to the cell surface depends on intact actin filaments [19], we and others have shown in more recent studies that M interacts with RNPs in viral inclusions and movement of viral RNPs to the plasma membrane occurs as co-transport with the M protein [5,20,21]. In agreement with the idea that actin filaments serve as tracks for movement of M-coated RNPs to the cell surface, disruption of the actin filaments resulted in an intracellular retention of both, M and RNPs. A very recent report proposes that interaction with the cytoskeleton is mediated by direct binding of F-actin to phenylalanine 50 in the M protein [11]. The direct interaction of MV M and actin is in line with findings for the M proteins of Newcastle disease virus and Sendai virus [22] that also serve as the recognition site for actin. However, it remains to be elucidated if M binds directly to actin, or if interaction is mediated via motor proteins. The latter might be supported by our finding that Jaspla treatment did not affect the M-RNP trafficking to the cell periphery. While movement along non-dynamic actin filaments must be assumed to be less efficient, motor proteins would still be able to transport cargo (M-RNPs) along stabilized actin filaments.

In contrast to many other viral matrix proteins [23], MV M protein does not possess any known late domain motif. Therefore budding does not depend on the cellular endosomal sorting complex required for transport (ESCRT) machinery [24]. Given that MV M-RNPs interact with the actin cytoskeleton and that the actin motor protein Myosin Vb interacts with members of the Rab11 family [25], a mechanism of late domain independent budding might be used by MV. The idea of a M-Rab11 interaction is indeed supported by the very recent finding that apical release from polarized epithelial cells depends on the Rab11A-dependent apical recycling endosomal pathway [26].

Multifunctional involvement of actin microfilaments during viral infection has been documented in many studies. Dependence of viruses on actin, however, differs drastically not only between different DNA and RNA virus families, but even between closely related virus family members. Thus, it must be concluded that each virus has evolved its own mechanism to interact with the cellular cytoskeleton machinery ensuring optimal replication. In contrast to our findings, HPIV-3 needs intact actin filaments for viral RNA synthesis [27]. Consequently, actin disruption led to a reduction of HPIV-3 release due to lack of viral proteins. For the budding process of HPIV-3, microtubules rather than actin filaments are important [28]. Since Cytochalasin B had no negative effect on VSV release, it was supposed that actin is not involved in VSV replication [29]. However, interaction of VSV M with dynamin was recently shown to be required for assembly [30]. Treatment of Rotavirus infected polarized epithelial cells with Jaspla did not reduce overall virus release but altered the budding polarity from apical to bipolar [13]. MV is also released apically from polarized epithelia [31-33], but actin treadmilling does not seem to play a role in polarized MV release since treatment of MV-infected polarized MDCK cells with Jaspla did not alter budding polarity (Dietzel, unpublished observation).

As in MV infection, disruption of the actin cytoskeleton reduced release and viral infectivity of HIV [34]. Recent cryo electron tomography studies have shown that HIV budding at the plasma membrane can be divided into different categories with respect to their actin context [35]. Even if most of the budding sites were found adjacent to filamentous actin, only half of them were associated with filopodia-like structures characterized by a parallel actin organization. The rest of the buds were found with cortical actin parallel to the plasma membrane, or with cortical actin directed towards or protruding into the budding site. Even if actin filaments have been shown to protrude into budding MV particles [8,18], one might speculate that highly pleomorphic MV particles also bud in different forms. Though we cannot rule out that the only partial block of virus release observed upon Jaspla treatment is due to an incomplete stabilization of actin filaments at the used concentrations, it might be speculated that only budding forms that have a distinctive requirement for actin dynamics or treadmilling are affected by cytocortical actin stabilization.

Recent observations have shown that the interaction of the MV glycoprotein complex with receptors on lymphocytes and dendritic cells (DCs) initiate cytoskeletal dynamics. In DCs, MV binding initiates host cell cytoskeletal dynamics needed for viral uptake and the establishment of functional synapses with T cells. Furthermore, MV binding to T cells causes a loss of polarization, adhesion and motility by actin cytoskeletal paralysis [36]. It is therefore highly likely that integrity and dynamics of actin filaments not only play an important role in virus maturation at the plasma membrane, but are also involved in MV-mediated immunosuppression.

## Conclusion

This paper demonstrates that intact actin filaments are required for M-RNP transport to the plasma membrane, and are thus needed to initiate assembly at the plasma membrane and to downregulate cell-to-cell fusion mediated by surface-expressed viral glycoproteins. We furthermore provide first conclusive evidence that actin dynamics are critically required in later steps in MV maturation, particularly for bud formation and the final pinching-off.

## Methods

### Cells and viruses

MDCK cells were cultured and infected in Minimal Essential Medium (MEM) supplemented with 10% fetal calf serum (FCS), Penicillin and Streptomycin. Vero cells were maintained in Dulbecco’s MEM supplemented with 10% FCS and antibiotics. Cells were infected during seeding with the MV Edmonston strain at an MOI of 10 (MDCK cells) or an MOI of 1 (Vero cells), respectively.

### Inhibitor treatment and virus growth analysis

Inhibitor stocks were prepared in DMSO at concentrations of 4 mM Cytochalasin D (Sigma-Aldrich) and 0.1 mM Jasplakinolide (Calbiochem), respectively. Different dilutions of the inhibitors were assessed (1–4 μM CD and 50–200 nM Jaspla) to determine appropriate working concentrations that have maximal effects on the actin cytoskeleton without causing a loss of cell viability. In all experiments, 4 μM CD were used. Jaspla was used in a concentration of 100 nM and 200 nM for MDCK and Vero cells, respectively. Inhibitors were diluted to final concentrations in cell culture medium with 2% FCS and added to infected cells at 12 h p.i.. To prevent syncytia formation in MV-infected cells, a fusion inhibitory peptide (FIP, Bachem) was added at a concentration of 0.1 mM [37]. Cell supernatants were taken at 24, 36, 48, 60 and 72 h p.i. for plaque titration. To determine cell-associated infectivity at 24, 48 and 72 h p.i., cells were scraped into OptiMEM (Invitrogen). After one freeze-thaw cycle using liquid nitrogen and a 37°C water bath, lysates were clarified by low-speed centrifugation, and the supernatant was used for plaque titration.

### Immunofluorescence analysis

Inhibitor-treated and control cells grown on Permanox ChamberSlides were immunostained at 48 h p.i.. To stain filamentous actin, cells were fixed with 2% paraformaldehyde (PFA) in DMEM for 15 min and subsequently permeabilized with 0.2% Triton X-100 in PBS for 10 min at room temperature. Filamentous actin was detected using 12.5 μg/ml Phalloidin-FITC (Sigma-Aldrich). M and N protein costaining was performed as described previously [5]. Briefly, cells were fixed and permeabilized with cold methanol/acetone (1:1) for 5 min. Cells were then incubated with a mouse monoclonal M-antibody (MAB8910, Millipore) and an N-specific polyclonal rabbit antiserum. Primary antibodies were detected using AF568- or 488-coupled secondary antibodies, respectively. For costaining of M and H, cell were fixed with 2% PFA and permeabilized with 0.2% Triton X-100. H protein was detected using a monoclonal antibody from mouse and an AF488-labeled secondary antibody. Subsequently, a saturation step with 5% mouse serum was performed. To stain the M protein, the M specific monoclonal antibody MAB8910 was labeled with AF555 using a Zenon labeling kit (Invitrogen) and added to the cells for 60 min on ice. Finally, cells were mounted in Mowiol and confocal fluorescence images were recorded using a Zeiss Axioplan2 LSM510.

### Surface biotinylation and western blot analysis

At 48 h p.i., infected and inhibitor-treated MDCK cells were surface biotinylated using S-NHS-Biotin (Calbiochem) as described earlier [3]. For actin, tubulin and MV-N staining, cell lysates were directly subjected to SDS-PAGE and transferred to nitrocellulose. Actin and tubulin were detected using specific mouse antibodies (Sigma-Aldrich, dilution 1:1000) and an AF680-coupled secondary antibody (Invitrogen, dilution 1:5000). N was detected using an N-specific rabbit polyclonal antiserum [5] at a dilution of 1:1000, and an IRDye800-conjugated secondary antibody (Biomol, dilution 1:5000). Residual supernatants were divided into three parts and used for immunoprecipitation of M, H and F. H was precipitated by K83 [3], F was precipitated with an Fcyt antiserum [38], and M was precipitated by incubation with MAB8910. After addition of 20 μl of a suspension of protein A-sepharose CL-4B (Sigma-Aldrich), immuncomplexes were washed and subjected to SDS-PAGE under reducing conditions. After blotting to nitrocellulose, M was detected using MAB8910 and an AF680-conjugated secondary antibody. Immunoprecipitated and surface-biotinylated H and F proteins were detected using Streptavidin-AF680. Labelled proteins were detected by the Odyssey infrared-imaging system (LI-COR).

### Cell transfections

MDCK cells were grown to 80% confluency and then cotransfected with pCG-MV H and pCG-MV F [39] using Lipofectamine 2000 (Invitrogen) according to the manufacturer’s protocol. Inhibitors and FIP were added in MEM 2% FCS at 6 h p.t. and cells were further incubated for 18 h (24 h p.t.).

### Fusion assays

MDCK cells were either infected with MV, or were cotransfected with MV-H and F in the presence of FIP, to prevent fusion. Inhibitors were added at the times indicated. At 48 h p.i. or 24 h post transfection, cells were detached by accutase (Sigma-Aldrich). Untreated Vero cells were also detached using accutase and mixed with MDCK cells at a ratio of 3:1 for infected, and 30:1 for transfected MDCK cells. Cocultures were incubated in DMEM 10% FCS for 5 h in the absence of FIP to allow fusion. Then, cells were fixed and stained by a 1:10-diluted Giemsa staining solution. Cell-to-cell fusion was quantified as described previously [40] by counting and averaging the number of nuclei of 20 randomly chosen syncytia.

### Electron microscopy

MV-infected cells were fixed for ultrastructural analysis cells by adding a 2× fixation solution containing 0.2 M PHEM [120 mM piperazine-N,N=−bis(2-ethanesulfonic acid) (PIPES), 50 mM HEPES, 4 mM MgCl2, 20 mM EGTA (pH6.9)], 8% PFA and 0.2% glutaraldehyde to the medium. After incubation for 30 min at room temperature, cells were scraped off and pelleted. The supernatant was discarded and 4% PFA in DMEM was added to the cell pellet. Subsequently, samples were processed as described previously [41]. Briefly, cells were postfixed for 60 min with 1% osmium tetroxide in 50 mM HEPES buffer (pH7.5). After washings, samples were stained overnight in a 2% aqueous uranyl acetate solution. Then, cells were dehydrated, and embedded in a mixture of Epon and Araldite. Ultrathin sections of the cells were stained with uranyl acetate and lead citrate and analyzed by using a JEM 1400 transmission electron microscope at 120 kV.

## Competing interests

The authors declare that they have no competing interests.

## Authors’ contributions

ED conceived the study, carried out all cell infections and inhibitor studies, analysed the samples and drafted the manuscript. LK performed the EM work, and edited the manuscript. AM conceived the study and drafted the manuscript. All authors read and approved the final manuscript.
